# First Report of CD4 Lymphopenia and Defective Neutrophil Functions in a Patient with Amebiasis Associated with CMV Reactivation and Severe Bacterial and Fungal Infections

**DOI:** 10.3389/fmicb.2017.00203

**Published:** 2017-02-13

**Authors:** Etienne Ghrenassia, Amélie Guihot, Yuan Dong, Pauline Robinet, Thierry Fontaine, Karine Lacombe, Thomas Lescot, Marie-Caroline Meyohas, Carole Elbim

**Affiliations:** ^1^AP-HP, Hôpital Saint-Antoine, Service des Maladies Infectieuses et TropicalesParis, France; ^2^Département d'Immunologie, AP-HP, Hôpital Pitié-SalpêtrièreParis, France; ^3^DHU FAST, CR7, Centre d'Immunologie et des Maladies Infectieuses, Sorbonne Universités, UPMC Univ Paris 06Paris, France; ^4^Institut National de la Santé et de la Recherche Médicale, U1135, Centre d'Immunologie et des Maladies Infectieuses-ParisParis, France; ^5^Institut National de la Santé et de la Recherche Médicale, Centre de Recherche Saint-Antoine, UMR-S 938Paris, France; ^6^Institut Pasteur, Unité des AspergillusParis, France; ^7^Unité de Réanimation Chirurgicale Digestive, Département D'anesthésie et de Réanimation Chirurgicale, AP-HP, Hôpital Saint-AntoineParis, France

**Keywords:** amebiasis, cytomegalovirus, aspergillosis, neutrophils, CD4 T cells

## Abstract

We report the case of a patient with acute necrotizing colitis due to invasive amebiasis associated with CD4 lymphopenia and impaired neutrophil responses. The course of the disease was characterized by CMV reactivation and severe and recurrent bacterial and fungal infections, which might be related to the decreased CD4 T cell count and the impaired functional capacities of neutrophils, respectively. The clinical outcome was positive with normalization of both CD4 cell count and neutrophil functions.

## Introduction

The patient, a 55-year-old man from Mali, was hospitalized in the internal medicine department of a general hospital in July 10, 2013 for febrile acute low back pain. His medical history was unremarkable. He came to France 30 years ago, and his last trip abroad was to Mali in April 2011. He worked in a restaurant as a kitchen porter. He smoked cigarettes, but did not drink alcohol or use narcotics. The patient reported asthenia, anorexia, weight loss (−21 kg; BMI 17), liquid stools once a day since several weeks. Laboratory finding included anemia with 6.4 g/dL hemoglobin, CRP at 157 mg/L, and a white blood cell count of 5.470/μL, with 83% neutrophils and 15% lymphocytes, as well as a CD4 T-lymphocyte count below 200/mm^3^ (Table 1). HIV serology was negative.

**Table 1:** **Absolute numbers of leukocytes, neutrophils, and lymphocytes, counted from fresh EDTA blood, with the Sysmex XE-500 Hematology Analyzer; percentages and absolute counts of CD3^+^ T cells, CD4^+^ T cells, CD8^+^ T cells, and CD19^+^ cells and percentages of CD8^+^ T-cell subpopulations were determined with CYTOSTAT tetraCHROME kits on a FC500 cytometer (Beckman Coulter) and on a Navios flow cytometer (Beckman Coulter)**.

	**Normal values [min–max]**	**Patient August 2013**	**Patient Dec 2013**	**Patient Feb 2014**	**Patient July 2014**
Leukocyte count	4,000–10,000	5,470	9,420	6,990	4,940
Neutrophil %	–	83	63	51	43
Neutrophil count (cells/mm3)	1,500–7,000	4,540	5,940	3,600	1,682
Lymphocytes %	–	15	17	33	41
Lymphocyte count (cells/mm3)	1,500–4,000	860	1,580	2,320	1,599
CD3^+^ T cells %	66–82	57	66	71	79
CD3^+^ T cell count (cells/mm3)	879–1,684	490	729	2,067	1,292
CD4^+^ T cells %	37–55	22	27	32	38
CD4^+^ T cell count (cells/mm3)	510–1,037	189	301	940	621
CD8^+^ T cells %	18–35	34	36	36	38
CD8^+^ T cell count (cells/mm3)	258–615	292	399	1,051	623
CD4/CD8 ratio	–	0.65	0.75	0.89	0.99
**CD8^+^ T CELL SUB-POPULATIONS**					
% CD45RA^+^CCR7^+^CD8^+^ (Naive)	_17−63_		_38_	_29_	49
% CD45RA^−^CCR7^+^CD8^+^ (Central Memory)	2–12		27	13	_18_
% CD45RA^−^CCR7^−^CD8^+^ (Effector Memory)	20–47		4	13	18
% CD45RA^+^CCR7-CD8^+^ (TEMRA)	7–40		31	44	26
% CD38^+^HLA-DR^+^CD8^+^	2–7		43	15	16
CD19^+^ cells %	10–21		7	14	6
CD19^+^ cell count (cells/mm3)	137–341		78	382	111

On July 12, the abdominal computed tomography (CT) showed a right retroperitoneum collection with a right colitis. No treatment was introduced until blood cultures were positive for group F *Streptococcus* on July 26–29. Then, antibiotic treatment with amoxicillin plus gentamycin was introduced.

Despite antibiotics he developed septic shock on August 02 and was transferred to the intensive care unit (ICU) department of the same hospital. The abdominal CT showed abscesses of the right kidney, right psoas, and liver, and thickening of the right colon, with loss of the contrast agent in the peritoneal cavity. A right hemicolectomy was performed. A new line of antibiotics was introduced (piperacillin-tazobactam, fluconazole, and amikacin). Orotracheal intubation and mechanical ventilation were needed. Cultures of abscess samples produced group F *Streptococcus* and *Escherichia coli*.

On August 8, a new septic shock occurred. CT showed abscesses of the liver, retroperitoneum, right kidney, right thigh, and psoas, associated with pneumoperitoneum linked to sigmoid perforation.

After beginning a new line of antibiotics (imipenem + amikacin), the patient was transferred to the ICU department at Saint-Antoine Hospital (Paris). Treatment required drainage procedures associated with a right nephrectomy and cholecystectomy. Cultures of the abscesses showed *E. coli, Staphylococcus epidermidis*, and *Bacteroides thetaiotaomicron*. Histological analysis of the right colon showed many ulcerations of the mucosa and submucosa, covered by many *Entamoeba histolytic*. Histological analysis of the right kidney showed many abscesses in which *E. histolytica* was present. Serology for *E. histolytica* was positive [Latex test: 1/32, positivity threshold >1/5; ELISA: 5 Arbitrary Unit (AU), positivity threshold> 1.1 AU].

On August 12, the diagnosis of acute necrotizing colitis due to invasive amebiasis was made. Treatment began with a systemic antiamebic agent (ornidazole) for a month and a local antiamebic (tiliquinol) for 2 weeks.

Moreover, histological analysis of an abscess in the right kidney showed three large cells with eosinophilic inclusion bodies suggestive of invasive CMV. Immunohistochemical analysis of this area found CMV antigen. In addition, quantitative PCR detected CMV DNA in blood (viral load: 20,900/ml) and in bronchoalveolar lavage fluid (viral load: 31,800/ml). Accordingly, treatment with ganciclovir began on August 12. ELISPOT-IFN-gamma assays were performed to evaluate CMV-specific T cells. Overlapping 15-mer peptides covering two CMV proteins serve as key target antigens for CMV-specific T cells: a late matrix protein (pp65) that is abundant during human CMV infection and an immediate early 1 (IE1) antigen protein that is indispensable for viral replication. CMV-specific T cells were mainly directed against pp65 but not against IE1 (data not shown); this finding suggests a non-protective immune response (Sacre et al., 2005).

On August 18, a new septic shock occurred. Abdominal CT showed new abscesses of the liver, retroperitoneum, right iliopsoas, and right thigh. A third surgery for abcess draining was performed, and antibiotherapy was enlarged to imipenem, vancomycin, ciprofloxacin. Furthermore, chest CT showed excavated nodules surrounded by ground glass opacity in both lungs. Macroscopic analysis of the newest abscesses revealed the presence of fungal filaments, and cultures were positive for *Aspergillus fumigatus*. *A. fumigatus* grew at 10E5 level in endotracheal aspiration. Moreover, the seric galactomanan elevated to 1.1 > 0.5.

These data indicated a diagnosis of confirmed invasive aspergillosis. The patient was then treated with voriconazole for 6 months.

Despite the severity of this condition, and despite 2 new episodes of ventilation associated pneumonia (VAP), documented with *Pseudomonas aeruginosa* and *Klebsiella pneumonia*, the condition of the patient improved. Tracheostomy was performed on September 13, to allow better ventilation weaning. Tracheostomy was finally removed on September 29 and patient was transferred in medical ward in November 4.

On December 28, 2013, the patient developed a last sepsis with spondylodiscitis due to *K. pneumoniae* producing extended-spectrum-beta lactamase, treated with association of meropenem and tigecyclin during 3 months. The succesion of infectious events is summarized in Table 2.

**Table 2:** **Temporal scheme summarizing the succession of infectious events characterized by clinical features, microbial analysis, and results of computed tomography (CT) together with the description of the different surgery and anti-infectious treatments that were applied through time**.

**Treatment**		**Amoxillin gentamycin**	**Piperacillin + tazobactum amikacin fluconazole**	**Imipenem amikacin**	**Imipenem amikacin ornidazole ganciclovir**	**Imipenem ciprofloxacin vancomycin ornidazole ganciclovir**	**Meropenem tigecyclin voriconazole**
Surgery			Abcess drain Right colectomy	Abcess drain Right nephrectomy Cholecystectomy Stomy for sigmoid perforation		Abcess drain	
CT	Right retroperitoneum abcess, night colitis		Several abcess in peritoneum, right retroperitoneum, liver. Right colon perforation	New abcess in right retroperitoneum, right kidney, right psoas, liver. Pneumoperitoneum		New abcess in right retroperitoneum, right psoas, right thigh, liver Lung nodules with groun glass halo	L2–L3 Spondylodiscitis Excavation of lung nodules
Microbial samples		Streptococcus F bacteriemia	Streptococcus F, *E. coli* in abcess Right colon: Many ulcerations and many *Entamobea histolytica* histolytica	*E. coli* Bacteroides thetaiotaomicron, Staphylococcus epidermidis in abcess Right kidney: Several abcess, *E. histolytica* histolytica	Blood PCR CMV: 20,900/mL	[-] Clostridium lnocuum and *Aspergillus fumigatus* in abcess [-] Blood PCR CMV: 1,800/mL [-] BAL PCR CMV:31,800/mL [-] Galactomanan 1.1 > 0.5 [-] Endotracheal aspiration: 10^5^ *Aspergillus fumigatus*	Blood culture: Klebsiella pneumoniae Disco-vertebral biopsy: Klebsiella pnegnaniae
Clinical features	Fever, weight loss, and diarrhea since several weeks	Persistant fever	Septic shock Endotracheal intubation Mechanical ventilation	New septic shock Mechanical ventilation		New septic shock Mechanical ventilation	Sepsis Spondylodiscitis
Time		07/12/2013	07/26–29/2013	08/02/2013	08/12/2013	08/18/2013	12/28/2013

Further immunological studies sought to understand these repetitive infections. Standard immunological studies (complement system, serum immunoglobulins) yielded normal results. No monoclonal peak was identified. Analysis of the CD4 and CD8 T-cell subpopulations demonstrated a low CD4/CD8 ratio, a normal CD8 cell count associated with a decrease in effector memory CD45RA^−^CCR7^−^CD8^+^ T cells and a concomitant increase in the CD45RA^−^CCR7^+^CD8^+^ central memory population, compared with levels in healthy controls. This disruption of CD8 homeostasis was associated with a marked increased percentage of CD8^+^ lymphocytes expressing the activation markers HLA-DR and CD38 (Table 1). Nevertheless, functional studies of T lymphocytes showed normal production of intracellular cytokines, i.e., IL-2 and IFNγ, after stimulation with CD3/CD28 and PMA (not shown).

**Figure 1:**
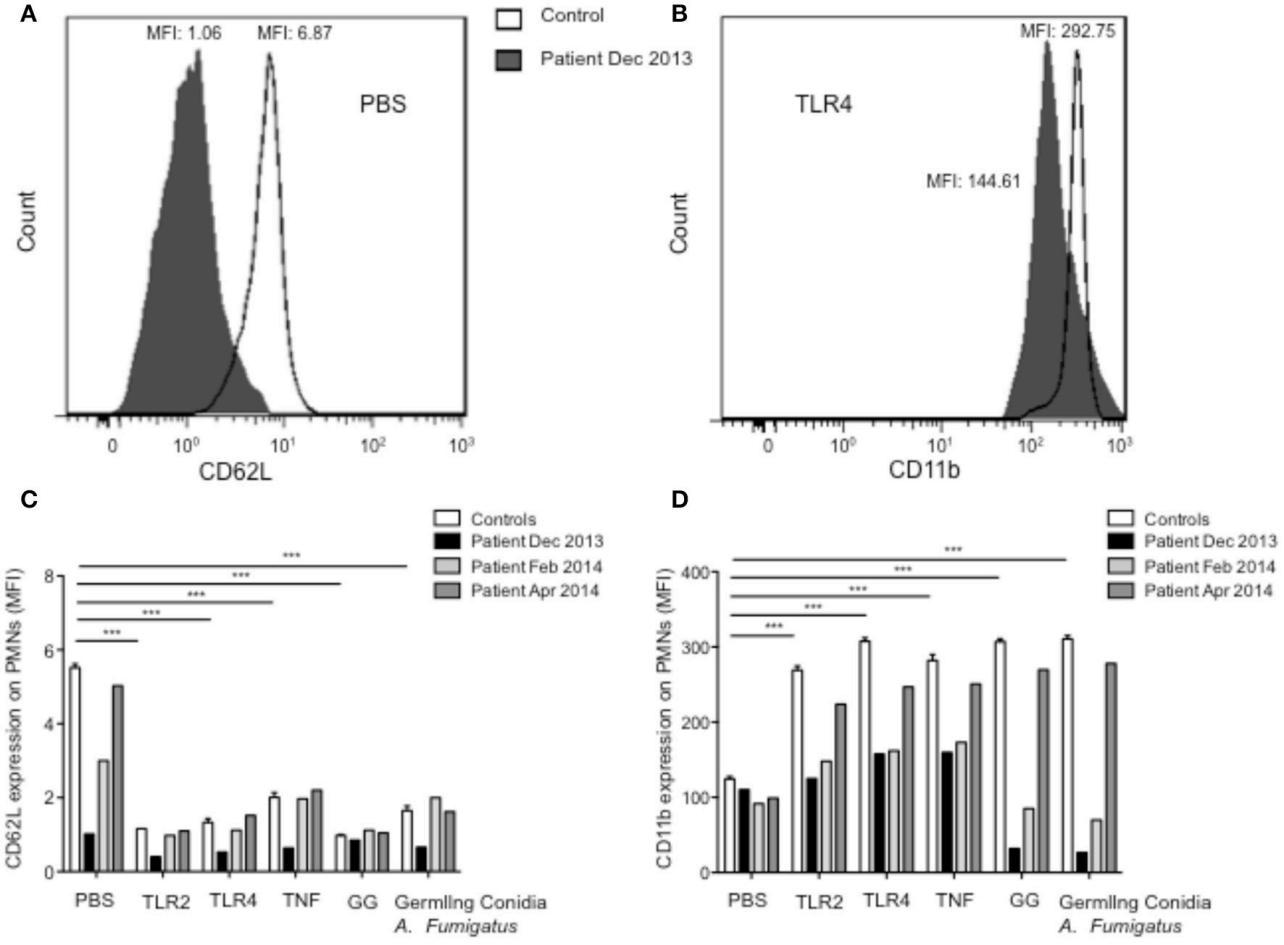
**Functional response capacity of PMNs from controls and patient. (A,B)** Expression of CD62L and CD11B on PMNs surface was measured after incubation of whole-blood samples at 37°C for 45 min with PBS. One histogram representative of CD62L **(A)** and CD11b **(B)** expression at the surface of PMNs from control (white) and patient Dec 2013 (black). Expression of CD62L **(C)** and CD11b **(D)** on PMN surfaces was measured after incubation of whole-blood samples at 37°C for 45 min with PBS, Pam_3_CSK_4_ (TLR1/2 agonist, 1 μg/ml), LPS (TLR4 agonist, 10 ng/ml), TNF (5 ng/ml), GG (20 μg/ml), or germling conidia from *A. fumigatus* (2 × 10^5^/ml). Samples were then stained with PE-anti-CD11b and APC-anti-L-selectin Abs at 4°C for 30 min. Results are expressed as mean fluorescence intensity (MFI). All measurements were performed in controls (*n* = 10, values are means ± sem) and in the patient at several different times. Each experiment in the patient was matched, at the same time, with an experiment in a healthy control with results in the normal range. Statistical significance determined by the nonparametric Mann–Whitney test is indicated. ^***^Significantly different from sample incubated with PBS *p* < 0.001.

**Figure 2:**
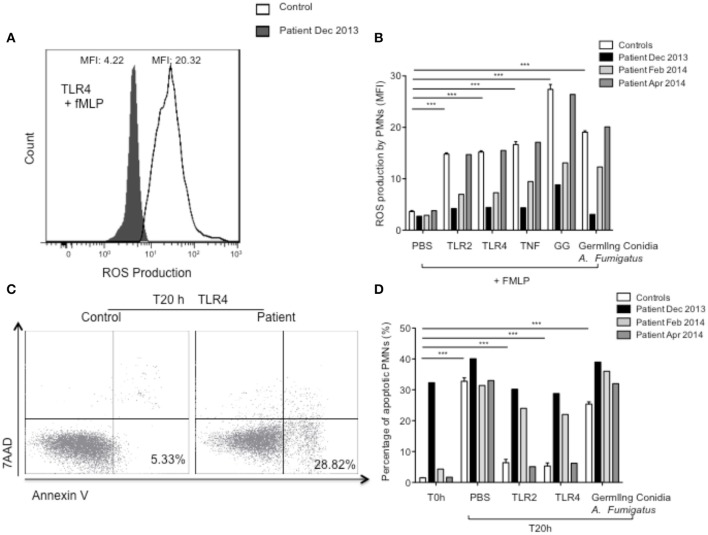
**ROS production and apoptosis of PMNs from controls and patient. (A,B)** ROS production by PMNs was studied by DHE oxidation; **(A)** ROS production by PMNs was measured after pretreatment of whole-blood samples for 45 min with TLR4 agonist and incubation for 5 min with fMLP (10^−6^ M). One histogram representative of ROS production by stimulated PMNs from control (white) and patient (Dec 2013) (black). **(B)** ROS production was measured after pretreatment of whole-blood samples for 45 min with PBS, Pam_3_CSK_4_ (TLR1/2 agonist, 1 μg/ml), LPS (TLR4 agonist, 10 ng/ml), TNF (5 ng/ml), GG (20 μg/ml), or germling conidia from *A. fumigatus*, and followed by fMLP (10^−6^ M, 5 min). Results are expressed as MFI. **(C,D)** Apoptosis was quantified by staining with annexin V and 7-aminoactinomycin-D. **(C)** PMN apoptosis was analyzed by incubating whole-blood samples in 24-well tissue culture plates at 37°C with 5% CO_2_ with TLR4 agonist. One cytogram representative of PMN death in control and in patient (Dec 2013). **(D)** PMN death was analyzed immediately after sampling (PMN spontaneous apoptosis, T0h) and by incubating whole-blood samples in 24-well tissue culture plates at 37°C with 5% CO_2_ with PBS, Pam_3_CSK_4_ (TLR1/2 agonist, 1 μg/ml), LPS (TLR4 agonist, 10 ng/ml), or germling conidia from *A. fumigatus* for 20 h at 37°C (PMN survival, T20h). PMNs were identified as CD15^high^ cells and 2 × 10^5^ events were counted per sample. Results are expressed as percentages of apoptotic cells. All measurements were performed in controls (*n* = 10, values are means ± sem) and in the patient at several different times. Each experiment in the patient was matched, at the same time, with an experiment in a healthy control with results in the normal range. Statistical significance determined by the nonparametric Mann–Whitney test is indicated. ^***^Significantly different from sample incubated with PBS *p* < 0.001.

In view of the severe recurrent and severe bacterial and fungal infections, we investigated neutrophil functions. Standard polymorphonuclear leukocyte (PMN) functional testing showed normal PMN migration under agarose and normal PMN chemiluminescence, respectively, ruling out leukocyte adhesion deficiency and chronic granulomatous disease. We then studied PMN responses elicited by toll-like receptor (TLR)-2, TLR4, TNFα, *A. fumigatus* germling conidia, and galactosaminogalactan (GG), a polysaccharide secreted by this fungus during early growth *in vivo* and reported to modulate immune response (Fontaine et al., 2011). As expected (Campillo-Gimenez et al., 2014), on stimulation, PMNs from controls shed L-selectin (CD62L) and increased their expression of the β2 integrin, CD11b/CD18. L-selectin was not detectable at the surface of unstimulated PMNs from the patient (Figure 1A), and after treatment of his sample with TLR2, TLR4, TNFα, GG, or *A. fumigatus* germling conidia (Figure 1C), CD11b increased moderately or not at all (depending on the stimulant), compared with unstimulated PMNs (Figures 1B,D). Also as expected (Campillo-Gimenez et al., 2014; Robinet et al., 2014), pre-treatment of whole blood from controls with TLR2, TLR4, or TNFα, or with GG or *A. fumigatus* germling conidia, followed by stimulation with fMLP, a structural analog of bacterial metabolic products, strongly increased the production of reactive oxidative species (ROS) by PMNs. Incubation of whole blood with the different agonists had little stimulatory effect on ROS production by the patient's PMNs (Figures 2A,B). We did not detect any antineutrophil cytoplasmic antibodies (ANCA), which have previously been associated with invasive amebiasis (Pudifin et al., 1994) and might impair PMN functional activity (Bartunková et al., 1997). Finally, immediately after sampling (T0h), the percentage of circulating apoptotic PMNs from controls was <2%, while for the patient it reached 32% (Figure 2D). In addition, incubation of whole-blood samples from controls with TLR agonists and TNF, rather than PBS, significantly decreased their percentage of apoptotic PMNs, as expected (Campillo-Gimenez et al., 2014; Robinet et al., 2014). Such PMN survival was not observed in the patient (Figures 2C,D).

Substantial improvement began within 3 months of initiating treatment with meropenem and tigecycline, for the spondylodiscitis in December 2013, and the patient' outcome was favorable. He was seen in consultation in July 2014 and June 2015. His general state was satisfactory; he has gained weight, returning to his baseline weight of 55 kg, and has had no infectious diseases. We have observed the progressive restoration of his CD4 count and homeostasis of the CD8^+^ T cells and PMN defects (Table 1, Figure 1). In addition, an ELISpot assay against CMV peptides showed a new positive response against IE1 (data not shown).

## Background

The enteric-dwelling protozoan parasite, *E. histolytica*, is the causative agent of amebiasis. Although, it is primarily a disease of underdeveloped countries, it may be found in travelers who have returned from endemic areas and ingested infective cysts through contaminated food or water. Compared with other parasites, the life cycle of *E. histolytica* is relatively simple and consists of 2 stages: the infectious cyst and the disease-inducing (motile) trophozoite stage. When amebic cysts are ingested via fecal contaminated food or water, they pass through the stomach and excyst in the terminal ileum where they mature into trophozoites and colonize the colon. About 90% of infections are asymptomatic (Haque et al., 2003). For unknown reasons, *E. histolytica* is capable of taking on a pathogenic phenotype. Disease occurs when trophozoites disrupt the mucosal barrier and penetrate the underlying tissue where they secrete enzymes that break down the extracellular matrix, destroy cells, and phagocytose cellular debris. After invading the mucosa and submucosa, trophozoites may enter portal circulation and disperse to the liver and other soft organs. Invasive disease includes dysentery and extra-intestinal amebiasis, most commonly amebic liver abscesses (ALAs), which occur in ~1% of symptomatic cases in developing countries. The host can mount both humoral and cell-mediated immune responses against *E. histolytica*, both of which are associated with protection. In patients with amebic colitis and ALA the parasite employs multiple strategies that allow it to successfully subvert the immune response and establish a chronic infection. In such cases the host develops a defective adaptive response incapable of clearing the parasite (Mortimer and Chadee, 2010; Nakada-Tsukui and Nozaki, 2016).

Sepsis is a descriptive term for a common disorder characterized by a broad and complex set of cellular changes evoked in response to infection or other signatures of danger. These changes are elicited by the engagement of conserved pattern recognition receptors including TLRs, NOD-like receptors (NLRs), RIG-I helicases, and C-type lectin receptors expressed on most cell types. They are ultimately effected through the expression or inhibition of a large number of immune and metabolic genes, and through post-translational changes in key intracellular proteins involved in signaling and transcriptional regulation. Following resolution of the primary infection, sepsis patients are susceptible to secondary infections and it has become clear that a sepsis-induced immunosuppressive state accounts for this increased vulnerability toward secondary infections (Koch et al., 2017). Secondary infections include opportunistic bacterial and fungal infections and viral reactivation, Pneumonia is the most common type of infection and Gram-positive bacteria most frequently involved are *Staphylococcus aureus* and *Streptococcus pneumoniae*, whereas *E. coli, K. pneumoniae*, and *P. aeruginosa* predominate among Gram-negative isolates. Bacteria-derived molecules like toxins degrade cell membranes resulting in upregulation of receptors targeted by bacteria for adhesion. This facilitates bacterial tissue invasion predisposing the host to secondary bacterial infections in a direct fashion. However, sepsis also renders the host more vulnerable toward secondary infections in an indirect fashion, by suppressing the immune system. In recent years, invasive Aspergillosis has also been recognized as an emerging disease of non-neutropenic patients in patients admitted to the ICU, even in the absence of an apparent predisposing immunodeficiency (Bassetti et al. 2014). Bacterial sepsis is also an associated trigger of CMV reactivation. Reactivation events associated with sepsis have been related to inflammatory stimulation of early promoter, transient relative immunoparalysis, and epigenetic regulation of viral DNA (Mansfield et al., 2015).

## Discussion

The amebic trophozoites develop multiple strategies to fend off the attack from elimination of immune cells. In particular, amebic trophozoites are able to kill a variety of cells, including T lymphocytes and neutrophils. *E. histolytica* is reported to kill T lymphocytes through an apoptotic process and to ingest apoptotic T cells via recognition of phosphatidylserine and collectins (Huston et al., 2000). In addition, a recent study reported that *E. histolytica* trophozoites ingested pieces of intact living T cells via trogocytosis. Killing and phago/trogocytosis of T cells could thus explained the decrease in CD4 T cells associated with a decrease in CD4/CD8 ratio which has been reported in patients with amebiasis associated with extraintestinal diseases such as liver abscess, with most patients recovering a normal CD4/CD8 ratio by one year after infection (Mortimer and Chadee, 2010). We can thus speculate that the decrease in our patient's CD4 T-cell count resulted from amebiasis. This decreased CD4 count might explain the reactivation of CMV that appeared in the course of amebiasis. Active CMV infection is a well-known opportunistic infection in immunocompromised patients, associated with a high viral load (Jaskula et al., 2009). CMV reactivation might also be related to the proinflammatory response associated with sepsis, as previously reported (von Muller et al., 2007; Cook and Limaye, 2012; Mansfield et al., 2016). Decrease in absolute number of all types of T cells has been reported in septic patients or septic shock, except the T regulatory cells. Thus, the decrease of CD4 count reported in the patient might also been related to septic conditions; nevertheless lymphopenia in sepsis is associated with a CD4/CD8 T cell ratio significantly elevated above normal range (Rimmelé et al., 2016).

At the peak of illness, the patient's CD45RA^−^CCR7^−^CD8^+^ T cell effector memory (TEM) count decreased at the same time as his CD45RA^−^CCR7^+^CD8^+^ central memory (TCM) population increased. The memory T-cell pool functions as a dynamic repository of antigen-experienced T lymphocytes that accumulate over the individual's lifetime. Reactive memory is mediated by TCMs that home to T-cell areas of secondary lymphoid organs, have little or no effector function, but readily proliferate and differentiate to effector cells in response to antigenic stimulation. TEMs migrate to inflamed peripheral tissues and display immediate effector function (Lanzavecchia and Sallusto, 2000). Decreases in the TEM subpopulation in the patient's peripheral blood may indicate that these cells migrated selectively out of the peripheral blood, or had a decreased survival rate, or that their differentiation from naive T cells to TCMs in peripheral blood was skewed. The disruption of peripheral blood homeostasis of CD8^+^ T cells, associated with an increase in the percentage of CD8^+^ lymphocytes expressing activation markers might reflect a persistent chronic inflammatory response, as previously reported in various pathological situations (Maldonado et al., 2003; Liu et al., 2007). In addition, septic conditions have been reported to substantially decrease the count of effector memory CD8 T-cells (Trgovcich et al., 2016).

PMNs play a very important role by combating bacterial and fungal infections. In response to pathogens, PMNs rapidly migrate from the blood to inflamed tissues where their activation triggers such microbicidal mechanisms such as the release of proteolytic enzymes and antimicrobial peptides, and the rapid production of ROS in oxidative burst (Nauseef and Borregaard, 2014). After killing microbes, PMNs die spontaneously, mainly by apoptosis; although they have a very short lifespan, their activation by circulating microbial products as well as by proinflammatory mediators promotes their survival and is a critical mechanism in their tissue accumulation and in their effectiveness against pathogens (Gabelloni et al., 2013). We analyzed the L-selectin (CD62L) and the β2-integrin CD11b/CD18 that play major roles in transendothelial migration, as well as ROS production in priming conditions; this ROS production is critical for bacterial killing. The normal increase in CD11b expression and ROS production in response to various stimuli were defective in the patient's PMN. In the patient, we observed increased PMN spontaneous apoptosis and a decreased survival in response to inflammatory mediators. These data might be related to the direct effect of *E. histolytica* on PMN apoptosis, through the activation of caspase-3 cascade, previously reported *in vitro* (Sim et al., 2005) and might explain at least in part the decreased expression of L-selectin at the PMN surface as well as impaired functional capacities of PMN in response to various stimuli (Kobayashi et al., 2005). These findings might thus explain the patient's severe and repetitive bacterial and fungal infections.

Sepsis is associated to a transient immunosuppression and septic patients are susceptible to secondary infections including opportunistic bacterial and fungal infections. Sepsis in humans is associated with a marked inhibition of rates of constitutive PMN apoptosis (Taneja et al., 2004; Rimmelé et al., 2016). PMNs are also capable of mediating crucial innate immune functions during sepsis. In particular, oxidative burst capacity of septic PMNs is intact (Drifte et al., 2013; Parlato et al., 2014; Rimmelé et al., 2016). Thus, given the major role of PMN against bacterial and fungal pathogens, it is highly likely that increased PMN apoptosis associated with impaired functional capacities of PMNs that we observed in the patient should be related to the direct effect of *E. histolytica* on PMN apoptosis and should be involved in the patient's increased susceptibility to bacterial and fungal infections. Of note, the first symptoms of the patient were weight loss and liquid stools; he developed a septic shock at a later stage. The initial sepsis due to *F Streptococcus* and *E. coli*, the plurimicrobacterial context, as well as the recurrence of septic shocks are highly suggestive of an altered gut integrity which, considering the context, might be related to the acute necrotizing colitis due to invasive amebiasis. In addition, although invasive aspergillosis has been reported in non-neutropenic and septic patients admitted to the ICU, it occurs mainly in patients with quantitative and/or qualitative defects of PMNs (Bassetti et al., 2014). Altogether, these data strongly suggest that invasive amebiasis might be the starting point of a new infectious burst.

## Concluding remarks

In conclusion, the case presented here enabled us to describe for the first time invasive amebiasis associated with CD4 lymphopenia and PMN defects. These later alterations could be related to the direct effect of *E. histolytica* on T lymphocytes and PMN apoptosis previously reported by *in vitro* studies. These abnormalities might be the starting point of a new infectious burst. In fact, the decreased CD4 count might explain, at least in part, the reactivation of CMV that appeared in the course of amebiasis. Impaired functional capacities of PMN in response to various stimuli might explain the patient's severe and repetitive bacterial and fungal infections.

## Ethics statement

All the clinical and paraclinic investigations were carried out only in the purpose of establishing a diagnosis and treating the patient and not in order to perform a research study. In France, such measurements that are vital for the patient and are part of a routine monitoring do not require an informed consent. The patient has given his consent for the publication of this manuscript.

## Author contributions

EG, KL, TL, and MM were involved in patient's recruitment and characterization, and summarized the patient's medical report. CE and AG designed the experiments. PR performed the experiments; TF prepared *A. fumigatus* germling conidia and galactosaminigalactan. AG, YD, MM, and CE contributed to data analysis. MM and CE wrote the manuscript with comments from co-authors. All the authors read and approved the final manuscript.

### Conflict of interest statement

The authors declare that the research was conducted in the absence of any commercial or financial relationships that could be construed as a potential conflict of interest. The reviewer WS and handling Editor declared their shared affiliation, and the handling Editor states that the process nevertheless met the standards of a fair and objective review.
